# The impact of BMI on subgroups of uterine cancer

**DOI:** 10.1038/sj.bjc.6605158

**Published:** 2009-06-30

**Authors:** K Lindemann, L J Vatten, M Ellstrøm-Engh, A Eskild

**Affiliations:** 1 Department of Obstetrics and Gynaecology, Akershus University Hospital, 1478 Lørenskog, Norway; 2 Department of Public Health, Faculty of Medicine, Norwegian University of Science and Technology, 7489 Trondheim, Norway; 3 Medical Faculty, Division of Akershus University Hospital, University of Oslo, 1478 Lørenskog, Norway; 4 Division of Mental Health, Norwegian Institute of Public Health, P.O. Box 4404 Nydalen, 0403 Oslo, Norway

**Keywords:** BMI, obesity, endometrial cancer, endometrioid adenocarcinoma, epidemiology

## Abstract

**Background:**

Obesity increases the risk of uterine cancer, but results by histological type have differed.

**Methods:**

We followed 36 755 women for 17.8 years for uterine cancers.

**Results and conclusion:**

Body mass index (BMI) was positively associated with uterine cancers as a whole, particularly for endometrioid adenocarcinomas, for which the relative risk for very obese women (BMI: ⩾40 kg m^−2^) compared with lean (BMI: 20–24 kg m^−2^) women, was 11.1 (95% confidence interval: 5.2–23.8).

The obesity epidemic contributes to a steadily increasing incidence of endometrial cancer for which obese women may have a six-fold higher risk compared with lean women (Lindemann *et al*, 2008). Most studies have assessed the effect on uterine cancers as a whole (Tretli and Magnus, 1990; Jonsson *et al*, 2003; Bjorge *et al*, 2007) or endometrial cancer without further differentiation (Brinton *et al*, 1992; Levi *et al*, 1992; Shu *et al*, 1992; Swanson *et al*, 1993; de Waard *et al*, 1996; Goodman *et al*, 1997; Schouten *et al*, 2006; Trentham-Dietz *et al*, 2006). However, it has been hypothesised that the positive association of obesity may be restricted to endometrioid adenocarcinomas, as this subtype appears to be oestrogen dependent, and oestrogen is produced in the adipose tissue (Siiteri, 1987; Azziz, 1989).

Two population-based studies have addressed the issue of whether obesity is differentially associated with histological subtypes of uterine cancer, but with inconsistent results. In one study, there was a stronger positive association with endometrioid adenocarcinoma (type I tumours) than for other subtypes (type II tumours, such as papillary, serous and clear cell adenocarcinomas, and some poorly differentiated carcinomas), as well as a weaker association for sarcomas and mixed endothelial and mesenchymal tumours (Bjorge *et al*, 2007). The other study differentiated between carcinoma and sarcoma variants, and found increased risk associated with obesity only for carcinomas (Tretli and Magnus, 1990).

In this prospective study of 36 755 women, we have compared the association of BMI with the risk of uterine cancer as a single entity, with all endometrial cancers, and with the risk of endometrioid adenocarcinomas.

## Materials and methods

Between 1984 and 1986, all inhabitants aged 20 years and older in the Nord-Trøndelag County in Norway were invited to participate in a study (the HUNT-I Study), in which standardised measurements of height and weight were made. A detailed description of the HUNT Study is given elsewhere (Holmen *et al*, 1991). Among the 38 260 women who participated, we excluded 1088 with prevalent cancer (except basal cell carcinoma) and 417 because their body mass index (BMI) could not be calculated. Thus, 36 755 women were followed up for cancer incidence.

Women diagnosed with cancer of the uterine corpus (International Classification of Diseases, 7th revision, ICD-7, code 172) were identified by the linkage of HUNT Study participants to the Norwegian Cancer Registry, using the unique 11-digit identity number of Norwegian citizens. Follow-up time was calculated as person-years from the HUNT baseline until the date of diagnosis of uterine cancer or any other cancer (except basal cell carcinoma), emigration, death or the end of follow-up, 31 December 2005, whichever occurred first.

The histological diagnoses are based on mandatory reporting to the Norwegian Cancer Registry from all pathological laboratories in Norway. Cancers diagnosed before 1993 were classified according to MOTNAC (Manual of Tumor Nomenclature and Coding). After 1993, tumours were classified according to ICD-O-2, a 6-digit code for morphology and grade of differentiation. According to the WHO classification (WHO Classification of Tumours, 2003), tumours were sub-classified as epithelial or mesenchymal tumours. Endometrioid adenocarcinomas included all variants (typical, villoglandular, with squamous differentiation, secretory and ciliated), and moderately or well-differentiated adenocarcinomas diagnosed before 1993.

Body mass index was calculated as weight divided by height squared (kg m^−2^), and categorised as <20, 20–24, 25–29, 30–34, 35–39 and 40 kg m^−2^ and higher. Information on prevalent diabetes (yes/no) and smoking status (current, former, never, missing) was obtained through self-administered baseline questionnaires. Information on recreational physical activity (i.e., walking, skiing, swimming or other sports) included the frequency (five categories), duration (four categories) and intensity (three categories) of the activity.

### Statistical analysis

We estimated age-adjusted relative risks (as hazard ratios) associated with BMI for different categories of uterine cancer using Cox regression analysis (SPSS statistical package, version 16.0, SPSS, Inc, Chicago, IL, USA). In three separate analyses, the following classifications were used all uterine cancers, all endometrial cancers and endometrioid adenocarcinomas. To estimate the log-linear trends of BMI, we included BMI as a continuous variable. In a separate analysis, we assessed BMI and the risk of uterine cancers other than endometrioid adenocarcinomas.

This study was approved by the Regional Committee for Ethics in Medical Research and by the Norwegian Data Inspectorate.

## Results

We followed up 36 755 women (mean age at baseline, 49 years) for an average of 17.8 years (range: 0–21). During follow-up, 263 histologically verified uterine cancers were diagnosed, and 224 of them (85%) were classified as endometrial cancers. Among the latter, 166 (74%) were classified as endometrioid adenocarcinomas (Table 1). In total 58 (26%) of the endometrial cancers were either other epithelial tumours (*n*=44/20%) or mesenchymal tumours (*n*=14/6%).

For uterine cancers as a whole, there was a strongly positive association with BMI (*P* for trend, <0.001) that was also present for endometrial cancers and for endometrioid adenocarcinomas (*P* for trend, <0.001). The relative risks across the categories of BMI were higher for endometrial cancers than for all uterine cancers, but confidence intervals (CIs) overlapped. Compared with the reference (BMI: 20–24 kg m^−2^), the age-adjusted relative risk for BMI ⩾40 kg m^−2^ was 8.3 (95% CI: 4.1–16.7) for endometrial cancer, 6.7 (95% CI: 3.4–13.4) for all uterine cancers and 11.1 (95% CI: 5.2–23.8) for endometrioid adenocarcinomas. For uterine cancers other than endometrioid adenocarcinomas (*n*=97), compared with the reference group (BMI: 20–24), there was an increased risk associated with a BMI of ⩾35 (age-adjusted relative risk 4.67 (95% CI: 2.4–9.2)), but there were no associations for the other BMI categories.

Further adjustment for prevalent diabetes, smoking and physical activity did not substantially influence the reported results (data not shown).

## Discussion

In this prospective study of 36 755 women, there was a strongly positive and linear association of BMI with the risk of cancer of the uterine corpus. Separate analyses showed that a stronger association of BMI with the risk of endometrioid adenocarcinomas than for uterine cancers as a whole. Previous studies have shown a two- to six-fold higher risk of uterine cancer among obese women compared with lean women. Most studies have used all uterine cancers as a single entity as the endpoint (Jonsson *et al*, 2003; Lindemann *et al*, 2008), or histologically verified endometrial cancers without further differentiation (Levi *et al*, 1992; Shu *et al*, 1992; Swanson *et al*, 1993; de Waard *et al*, 1996; Goodman *et al*, 1997; Schouten *et al*, 2006; Trentham-Dietz *et al*, 2006). The results related to the histological subtypes of uterine cancer have been inconsistent; one study reported positive associations of BMI with the risk of endometrioid adenocarcinomas (type I tumours) and papillary, serous and clear cell adenocarcinomas, as well as sarcomas and mixed tumours (Bjorge *et al*, 2007). The other study distinguished between carcinomas and sarcomas, and found that BMI was positively associated only with uterine carcinomas (Tretli and Magnus, 1990).

A weakness of our study is the incomplete histological classification of endometrial cancer subtypes before 1993 when the classification of uterine cancer in the Norwegian Cancer Registry was changed, allowing more refined analyses. Thus, 111 (64%) of the endometrioid adenocarcinomas were diagnosed after 1993, when the classification distinctly differentiated between type I, type II and other tumours of the endometrium. In our study, 74% (*n*=166) of all endometrial cancers were classified as endometrioid adenocarcinomas, which accords with the literature (Sherman, 2000). Another limitation is the lack of control for reproductive factors, such as parity, the use of oral contraceptives and postmenopausal hormone therapy. Generally, Norwegian women were restrictive in their use of hormone therapy in the 1980s, but this subsequently increased to ∼35% of women in their postmenopausal stage in the 1990s. However, the use of combined oestrogen–progesterone preparations, which constituted 70% of the medication, has not been associated with increased risk of endometrial cancer in Norway (Bakken *et al*, 2004).

The increased risk associated with obesity has been attributed to higher concentrations of endogenous oestrogen hormones. Oestrogens produced in the adipose tissue have a direct mitogenic effect on endometrial cells, and it is assumed that this effect is not counterbalanced by progesterone because of chronic anovulation accompanied by reduced progesterone synthesis. However, weight-related increase in endometrial growth factors (Pasquali *et al*, 1997), cytokines (i.e., leptin, adiponectin) (Petridou *et al*, 2002; Housa *et al*, 2006) or transcription factors may be related to the development of uterine tumours (Roberts-Thomson, 2000).

Our results suggest a positive association of BMI with all subtypes of uterine cancer, but the association was strongest for endometrioid adenocarcinomas. However, the moderate precision of the estimates warrants that future studies assess how obesity is related to different subtypes of uterine cancer.

## Figures and Tables

**Table 1:** Relative risk (RR) of cancer of the uterine corpus according to histological subtype in the study population of 36 755 women in Norway

		**Uterine cancers (*n*=263)**			**Endometrial cancers (*n*=224)**			**Endometrioid adenocarcinomas (*n*=166)**		
**Variable**	**No. of persons**	**RR^a^**	**95% CI**	***P* for trend**	**RR^a^**	**95% CI**	***P* for trend**	**RR^a^**	**95% CI**	***P* for trend**
*BMI*										
<20	3065	0.7	0.3–1.5		0.6	0.2–1.5		0.8	0.3–1.9	
⩾20–24	17 967	1.0			1.0			1.0		
⩾25–29	10 820	1.6	1.2–2.2		1.8	1.3–2.4		2.1	1.4–3.0	
⩾30–34	3685	2.0	1.3–2.9		2.1	1.4–3.2		2.1	1.3–3.5	
⩾35–39	972	5.3	3.4–8.2		5.6	3.5–9.1		5.8	3.3–10.3	
⩾40	246	6.7	3.4–13.4	<0.001	8.3	4.1–16.7	<0.001	11.1	5.2–23.8	<0.001

BMI=body mass index; CI=confidence interval.

a Adjusted for age in 10-year categories (<30, 30–39, 40–49, 50–59, 60–69, ⩾70).
